# Assessment of clonal fidelity of *Tylophora indica* (Burm. f.) Merrill “*in vitro*” plantlets by ISSR molecular markers

**DOI:** 10.1186/2193-1801-3-400

**Published:** 2014-08-03

**Authors:** Madan Mohan Sharma, Roop Narayan Verma, Abhijeet Singh, Amla Batra

**Affiliations:** Plant Biotechnology Laboratory, Department of Botany, University of Rajasthan, Jaipur, India

**Keywords:** Clonal fidelity, ISSR markers, Organogenic calli, *Tylophora indica*, UPGMA

## Abstract

*Tylophora indica* Burm F. Merrill. is widely used against various diseases owing to the presence an array of medicinally important secondary metabolites. Its stem is bitter, stomachic, stimulates bile secretion, enriches the blood and cures diseases like diabetes, fever, flatulence, hypertension, jaundice, leucorrhoea, urinary disease and upper respiratory tract infection. It is neglected for tissue culture work because of deciduous nature of climbing shrub, facing problems for micropropagation. Hence, *in vitro* regeneration of complete plantlets was done through indirect organogenesis in *Tylophora indica*. Calli were produced from *in vivo* leaves of *T. indica* on MS medium supplemented with 6-Benzylaminopurine (BAP: 2.0 mg l^-1^) and Indole-3-butyric acid (IBA: 0.5 mg l^-1^). The multiple shoots (12.00 ± 1.50) emerged and elongated on MS medium fortified with Thidiazuron (TDZ: 0.1 mg l^-1^). They were rooted on half strength MS medium having IBA (0.5 mg l^-1^) (7.75 ± 0.25) after 20 days of sub-culturing followed by hardening and acclimatization. During indirect regeneration of plants, chances of somaclonal variations may arise. These variations should be identified to produce true to type plants. Plantlets raised through tissue culture were used to validate the clonal fidelity through Inter simple sequence repeat markers (ISSR). Clonal fidelity is a major consideration in commercial micropropagation using *in vitro* tissue culture methods. During the study, total 71 clear and distinct bands were produced using 6 primers. The banding pattern of each primer was uniform and comparable to mother plant and showed about 93% homology using un-weighted pair group method with arithmetic averaging (UPGMA). ISSR analysis confirmed the genetic stability of *in vitro* raised plants.

## Introduction

*Tylophora indica* Burm F. Merrill., is a perennial and branching climber indigenous to India and native to the plains, forest and hills of southern and eastern India and grows up to an attitude of 1,000 m in Bengal, Assam, Cachar and Orissa (Ali 2008). It belongs to family Asclepidaceae and is commonly known as Indian Ipecacuanha (Bhavan 1992). Traditionally, it is used as a folk medicine in various regions of India for the treatment of bronchial asthma, bronchitis, allergies, rheumatism and dermatitis (Butani et al. 2007). Besides, it is also a good remedy for anti-psoriasis, seborrheic, anaphylactic, leucopenia and as an inhibitor of the Schultz-Dale reaction (Sarma and Misra 1995). Leaves and roots have medicinal potency and are recognized to show laxative, expectorant, diaphoretic and purgative properties. Tylophorine and tylophorenine are major alkaloids in this plant, which are responsible for a strong anti-inflammatory action (Rao et al. 1971; Gopalakrishnan et al. 1979).

The rate of plant propagation is critical to meet the pharmaceutical demand for Tylophorine, an antiasthamatic drug. A slow propagation rate has been reported in *T. indica*, because of low seed viability, poor germination rate, rare fruit set as well as a small number of propagules (stem cuttings), has restricted the natural dissemination of the plant (Thomas and Philips 2005). In addition, the destruction caused by harvesting the roots and leaves as a source of the drug has threatened the survival of the plant (Faisal and Anis 2007). Thus, *in vitro* techniques have been applied for large scale production.

During plant tissue culture, genotypic variation can be induced at any stage of development of plantlets. Earlier, genetic variations have been confirmed using a variety of molecular markers in *Gossypium hirsutum* (Jin et al. 2008), *Bambusa balcooa* (Negi and Saxena 2010), *Nothapodytes foetida* (Chandrika et al. 2010), Musa spp. (Lu et al. 2011), *Oryza sativa* (Shan et al. 2012), Spilanthes calva DC (Razaq et al. 2013), Moringa peregrina (Forsk.) (Al Khateeb et al. 2013) and *Pilosocereus robinii* (Khattaba et al. 2014).

Consequently, a rigorous analysis of genetic stability of the plantlets produced through tissue culture becomes essential. ISSRs are DNA fragments of about 100–3000 bp located between adjacent, oppositely oriented microsatellite regions. They are dispersed throughout the genome and vary in the number of repeat units. To ascertain genetic variations in number as well as sizes of repeat units, we produced *in vitro* plants of *T. indica* through callus culture and detected genomic variation through reliable, reproducible, low cost and successful molecular markers technology i.e. ISSR which are useful in areas of genetic diversity, phylogenetic studies, gene tagging, genome mapping and evolutionary biology in a wide range of crop species.

The present study was conducted with an aim of ascertaining the genetic uniformity of the tissue culture raised plantlets with respect to mother plant- *T. indica using* ISSRs markers.

## Material and methods

### Plant material

The experiments were conducted to initiate and produce stock callus through leaf explants for further morphogenetic differentiation. Leaf explants were separately inoculated on MS medium fortified with different concentrations of BAP (0.5-3.5 mg l^-1^) to initiate callusing. It was followed by subculturing on MS medium containing various concentrations of BAP (0.5-3.5 mg l^-1^) with IBA (0.025-3.0 mg l^-1^), 1-Naphthaleneacetic acid (NAA: 0.5-5.0 mg l^-1^) and 2,4-Dichlorophenoxyacetic acid (2,4-D: 0.5- 5.0 mg l^-1^) for the production of stock calli. Subsequently, the small clumps of calli were subcultured for emergence of shoots. *In vitro* regenerated shoots were rooted on half strength MS medium supplemented with IBA (0.05-0.5 mg l^-1^) and indole-3-acetic acid (IAA: 0.05-0.5 mg l^-1^). Regenerated plantlets were hardened and acclimatized. These regenerated plantlets were used to procure leaf explants for ISSR analysis. However, four samples of *T. indica* were used during the studies from Department of Botany, Rajasthan University, Jaipur (T_1_) as mother plant, *in vitro* raised plant produced from T_1_ mother plant (T_2_), World Arboretum, Jhalana Dungari, Jaipur (T_3_) and Kulish Smriti Van, Jaipur (T_4_).

### Isolation and quantification of genomic DNA

Healthy leaves washed with running tape water for about 15 min. and wiped with tissue paper used of isolation of genomic DNA. *In vitro* leaves did not require surface sterilization. Further, 3.0 gm of each leaf sample was homogenized. The homogenized material proceeded by CTAB method (Doyle and Doyle 1990) modified by Sharma et al*.* (2003). 3.0 gm of leaves were grounded in chilled methanol with autoclaved mortar-pestle. The homogenized material was transferred to 15 ml pre-warmed (60°C) DNA Isolation Buffer (2X CTAB DNA Extraction Buffer-100 mM Tris, 20 mM EDTA, 1.4 M NaCl, 2% CTAB and 2 μl/ml β-mercaptoethanol) in capped polypropylene tubes (PP). The clump of homogenized material was suspended using spatula. The content was incubated for 45 min at 60°C and mixed to make solution homogenate by gentle swirling in water bath for 2–3 times. After removing from water bath 15 ml of chloroform: isoamyl alcohol (24:1) was added and mixed by inversion for 30 min to ensure emulsification of the phases. Content was centrifuged at 15000 rpm for 15 min at 4°C (Remi C 24 plus). Aqueous phase was taken and transferred to another polypropylene tube. Ice cold 6.0 ml of isopropanol was added, gently swirled and kept in deep freezer at -20°C for 30 min to precipitate DNA. The precipitated DNA-CTAB complex was collected by centrifuging the material at 15000 rpm for 15 min at 4°C. Supernatant was discarded and and washed with 70% alcohol and kept for 20 min with gentle agitation. The pellet was washed by centrifuging the tube at 10,000 rpm for 10 min and 4°C. The liquid was discarded and above step was repeated twice to remove all impurities. The tubes were inverted and drained on a paper towel. The pellet was dried over-night after covering with parafilm with tiny pores. The pellet was re-dissolved in 100 μl of TE buffer by keeping over night at room temperature without agitation.

### Purification of DNA

RNA was removed by treating the sample with DNase free RNase enzyme. Protein including RNase was removed by treating with chloroform: Isoamyl alcohol (24:1). 2.5 μl of RNase was added to 0.5 ml of crude DNA extract. It was mixed gently but thoroughly and incubated at 37°C for 1 hr. After 1 hr, a mixture of 0.3 - 0.4 ml of chloroform: Isoamyl alcohol (24:1) was added and mixed thoroughly for 15 min till an emulsion was formed. The emulsion was centrifuged at 15000 rpm for 15 min at 4°C. Supernatant was taken by avoiding the whitish layer at interface. The DNA was re-precipitated by adding double quantity of ice cold absolute alcohol. To pellet the DNA, the tube was centrifuged for 5 min at 10,000 rpm. The pellet was washed with 70% alcohol and dried over night. The DNA was re-dissolved in 100 μl of TE buffer.

### Quantification of DNA

The quantification of DNA was done at 260 nm & 280 nm UV in spectrophotometer (Optigen 2020 plus UNICAM). The quantitated DNA was diluted to final concentration of 25 ng/μl in TE buffer (10 mM Tris Cl, 1 mM EDTA, pH 8.0). The diluted DNA was again quantified and confirmed for accurate measurement on 0.8% agarose gel. All the diluted DNA samples were ranged from 23–27 ng/μl.

### PCR reactions

For ISSR analysis, 25 primers (set # 9) were screened followed by four PCR reactions. The PCR consisted of an initial denaturation step at 94°C for 5 min, 40 cycles comprising denaturation cycle at 94°C for 30 sec., annealing at 43°C for 30 sec., and extension at 72°C for 1 min and a final extension step at 72°C for 7 min. followed by hold at 4°C. PCR amplifications were performed on a DNA thermocylcer (Mycycler, BioRad).

### ISSR Primer selection

25 oligo-nucleotide primers (set # 9) obtained from the Genomic Chemical Cooperation, University of British Columbia, Vancouver, Canada were screened. Finally, 6 anchored ISSR primers for *T. indica* (Table 1) were used for the analysis of genetic diversity of 4 genotypes of the plant.

**Table 1:** **Details of primers, bands and polymorphism in** ***T. indica*** **revealed by 6 primers used in ISSR–PCR**

Primers	Sequences	Total No. of bands (a)	Total No. of Polymorphic bands (b)	Polymorphism (b/a*100)	Total No. of monomorphic bands (c)	Monomorphism (c/a*100)	Average PIC
	(5’→3’)						
807	AGAGAGAGAGAGAGAGT	8	7	87.50	1	12.50	0.5
812	GAGAGAGAGAGAGAGAA	16	6	37.500	10	62.50	0.82812
825	ACACACACACACACACT	12	4	33.333	8	66.66	0.875
827	ACACACACACACACACC	11	11	100.000	0	0	0.56818
840	GAGAGAGAGAGAGAGAYT	5	3	60.000	2	40	0.7
846	CACACACACACACACART	19	10	52.632	9	47.36	0.71052
Total		71	41	57.74	30	42.26	0.57

*= Multiply.

### Annealing temperature for ISSR primers

Annealing temperature for every primer was estimated using gradient PCR and optimum one was selected on the basis of sharpness and reproducibility of bands.

### Reproducibility of amplification patterns

DNA amplifications with each ISSR primer were repeated at least twice to ensure reproducibility. The bands were considered reproducible and scorable only after observing and comparing them in two separate amplifications for each primer. Clear and intense bands were scored while faint bands against background smear were not considered for further analysis.

## Results and discussion

**Figure 1:**
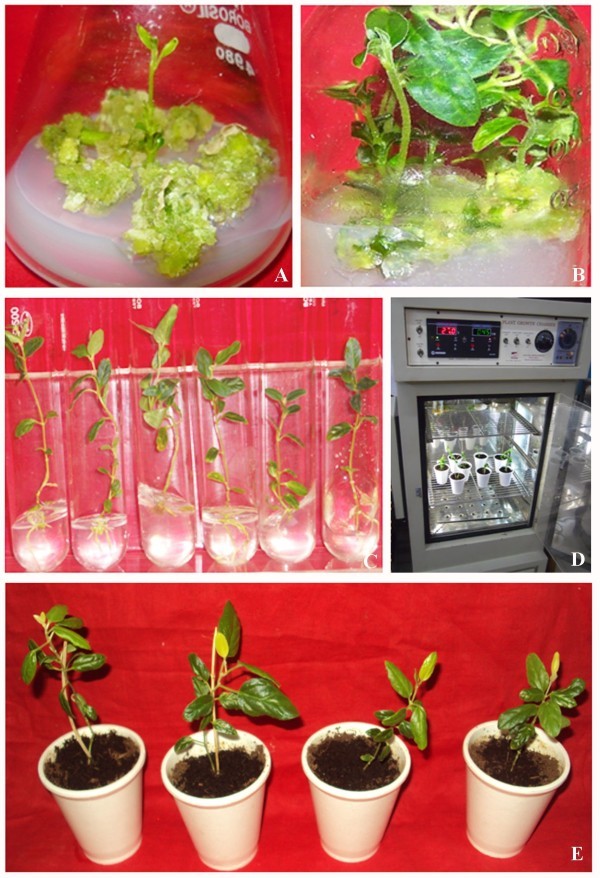
**Indirect regeneration of** ***Tylophora indica*** **. A**. Callus induced from the leaf and further regeneration of shoots. **B**. Shoot organogenesis from callus on MS medium along with BAP (2.0 mg l^-1^) and IBA (0.5 mg l^-1^). **C**. *In vitro* elongation of shoots on TDZ (0.1 mg l^-1^) and further root induction on ^1^/_2_ MS medium with IBA (0.5 mg l^-1^). **D**. Hardening in the plant growth chamber. **E**. Hardened plants in thermocol cups.

During the experimentation, leaf (3–5 mm^2^) segments cultured on MS medium augmented with BAP (2.0 mg l^-1^) and IBA (0.5 mg l^-1^) proved to be optimum for stock callus production after 3 weeks of inoculation (Figure 1A, B) (Table 2). Similar results were also noticed in *Melia azedarach* L. (Vila et al. 2004). In contrast, Lin et al*.* (2009) reported that Kn is better than BAP to induce callusing from leaf explants in case of *Ocimum santum*. Complete plantlet regeneration via indirect shoot organogenesis has been achieved from the culture of leaf explants. The multiple shoots (12.00 ± 1.50) were emerged from callus clumps (Figure 1B) (Table 2). At the same time, callus mediated shoot morphogenesis has been well documented in several medicinal plants such as *Tylophora indica* (Faisal and Anis 2005; Dennis and Boban 2005), *Saussurea obvallata* (Dhar and Joshi 2005), *Euphorbia nivulia* (Sunandakumari et al. 2005) and *Cassia angustifolia* (Agrawal and Sardar 2006). IBA (0.5 mg l^-1^) incorporated in half strength of MS medium induced optimum rooting (7.75 ± 0.25) without formation of callus at the cut end of shoots (Table 3). However, IAA at its all concentrations used did not produce promising results. *In vitro* emerged roots on IBA (0.5 mg l^-1^) were elongated on MS medium supplemented with TDZ (0.1 mg l^-1^) (Figure 1C). These *in vitro* regenerated plantlets were hardened and acclimatized in the field conditions with 75% survival rate (Figure 1D-E). At the same time, IBA also proved to be optimum for *in vitro* root induction in various plant species such as *Phyllanthus amerus* Shum. and Thonn (Sen et al. 2009), *Murraya koenegii* L. Spreng. (Rani et al. 2012), *Mentha arvensis* (Shahzad et al. 2002) and *Hoya wightii* spp. Palniensis (Lakshmi et al. 2010). However, in oppugnance to the above results, Dhabhai et al*.* (2010) in *Acacia nilotica* and Escutia et al*.* (2010) in *Tigridia pavouia* (L.F.) DC found NAA to be optimum for rooting.

**Table 2:** **Effect of plant growth regulators for callus induction, their further proliferation into multiple shoots through leaf explants**

Plant growth regulators (mg l ^-1^)				Leaf derived calli	
BAP	NAA	2,4-D	IBA	% response	Number of shoots/explant*Mean ± S.E. t _0.05_
0.5				20	2.25±0.10
1.0				35	2.88±0.50
1.5				40	3.15±0.22
2.0				48	6.11±1.50
2.5				46	5.50±0.40
3.0				30	4.90±0.10
3.5				23	3.10±0.20
2.0	0.5			19	2.20±0.10
2.0	1.0			22	2.45±0.15
2.0	2.0			35	2.75±0.25
2.0	3.0			37	3.55±1.50
2.0	4.0			28	3.40±0.40
2.0	5.0			16	2.90±0.10
2.0		0.5		9	0.90±1.05
2.0		1.0		12	1.15±0.22
2.0		2.0		20	1.91±0.54
2.0		3.0		31	3.15±0.42
2.0		4.0		24	2.56±0.56
2.0		5.0		21	2.14±0.15
2.0			0.025	80	11.14±0.90
2.0			0.5	95	12.00±1.50
2.0			1.0	75	9.28±1.00
2.0			2.0	68	8.75±0.80
2.0			3.0	55	6.45±0.25

*= Multiply.

**Table 3:** **Influence of half strength of MS salts along with various auxins on** ***in vitro*** **rooting response**

Plant growth regulators (mg l ^-1^)		% response of rooting	No. of roots per cuttings	Days taken for the emergence of roots
			*Mean ± S.E. t _0.05_	
IBA	IAA			
0.05		10%	1.10±0.41	15-16
0.1		28%	2.25±0.24	12
0.2		35%	3.14±0.56	11-12
0.3		45%	4.45±0.58	11
0.4		72%	6.65±0.85	10
0.5		85%	7.75±0.25	20
	0.05	5%	1.05±0.10	14-16
	0.1	15%	1.08±0.51	13
	0.2	25%	2.10±0.65	12-13
	0.3	45%	4.45±0.52	11
	0.4	35%	3.35±0.54	12
	0.5	22%	2.36±0.26	13

*= Multiply.

Genetic diversity in many plant species can be determined using morphological characteristics; isozyme and DNA based markers. DNA-based markers provide powerful and reliable tools for discerning variations within crop germplasm and for studying evolutionary relationships (Gepts 1993).

ISSR markers are the direct reflection of abundances and distribution of micro satellite repeat in the genome. In the present study, all possible combinations of dinucleotide repeats were included to find out most suitable characterization of the plant genome, because ISSR has already been used in numerous organisms for genetic characterization (Reddy et al. 1999), to assess genetic diversity (Zhang et al. 2006; Wang et al. 2012; Djamila et al. 2012; Yadav et al. 2013; Phulwaria et al. 2013), to identify genetic trait loci (Arcade et al. 2000; Guasmi et al. 2012; Najaphy et al. 2012) and for understanding phylogenetic relationships (Wolfe and Randle 2001; Datwyler and Wolfe 2004; Wu et al. 2005). ISSR amplification is a PCR based method that can rapidly differentiate closely related individuals (Zietkiewicz et al. 1994). This technique involves amplification of DNA segment between two identical microsatellite repeat regions.

Out of 25 primers screened, only 6 primers produced amplification. Finally, 6 anchored ISSR primers were used for the analysis of genetic diversity amongst 4 samples. Six dinucleotide repeat based ISSRs primers (807, 812, 825, 827, 840, and 846) in *T. indica* were amplified by PCR. AT/TA repeat based ISSRs were not amplified in the study, while maximum band positions and contrasting banding patterns were obtained using CT/CA/GA/AG/AC repeat based ISSRs (Table 1).

The results of amplified fragments, specific markers for each accession of *Tylophora indica* using ISSR-PCR analysis were observed and compared with other samples. For each ISSR marker, total amplified bands, total number of monomorphic bands, total number of polymorphic bands, percentage of polymorphic bands, percentage of monomorphic bands and polymorphism information content (PIC) were calculated. Six primers produced a very high degree of polymorphism with a total of 71 reproducible fragments ranging from 3 (primer 840) to 11 fragments (primer 827). The results showed that 30 bands were monomorphic and 41 amplified bands were polymorphic. The highest percentage of polymorphism (100%) was recorded using the primer 827, while the lowest percentage (33.33%) was recorded using primer 825.

**Figure 2:**
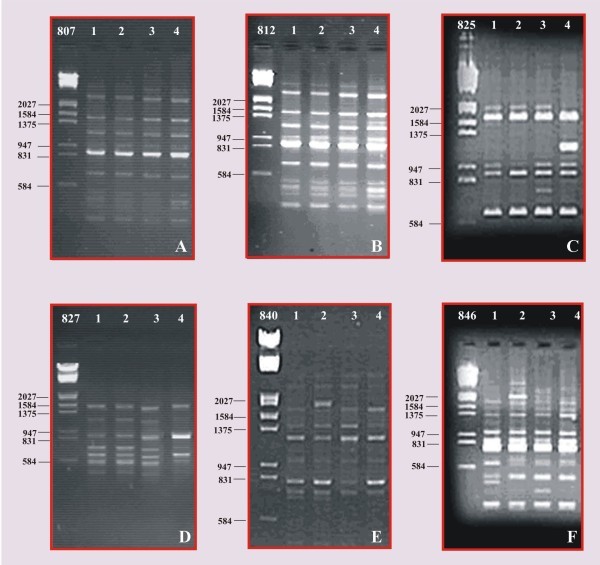
**ISSR products of** ***T. indica*** **amplified along with marker**  **DNA/EcoR I-Hind III. A**. ISSR primer- 807. **B**. ISSRprimer- 812. **C**. ISSRprimer- 825. **D**. ISSRprimer- 827. **E**. ISSRprimer- 840. **F**. ISSRprimer- 846.

The representative profile of the four accessions and the control with six primers has been shown (Figure 2A-F). The size of polymorphic fragments with six primers varied from 202 to 2125 bp and the number of amplified products ranged from 3 to 11 (Table 4).

**Table 4:** **DNA bands of Tylophora accessions compared with primers**

Primer	Size (bp)	T _1_	T _2_	T _3_	T _4_	Primer	Size (bp)	T _1_	T _2_	T _3_	T _4_
807	676	0	1	1	1	827	302	1	1	1	0
	714	1	0	0	0		554	1	1	1	0
	857	1	1	1	1		584	1	1	0	0
	1140	0	0	0	1		634	0	0	1	0
	1185	1	1	1	0		654	1	1	0	1
	1375	0	0	0	1		743	1	1	1	0
	1398	0	0	1	1		831	0	0	1	0
	1421	1	0	0	0		878	1	1	0	1
812	240	1	1	1	1		1248	1	1	0	0
	313	0	1	1	1		1534	0	0	1	0
	396	1	1	1	1		1584	1	1	0	1
	441	1	1	1	1	840	724	1	1	1	1
	662	1	1	1	1		802	1	1	0	1
	801	1	1	1	1		1201	1	1	1	1
	845	1	1	1	1		1225	0	1	1	0
	916	0	1	1	1		1904	0	1	0	0
	947	1	1	1	1	846	202	1	1	1	1
	1237	0	1	1	1		318	0	0	1	0
	1242	1	1	1	1		408	1	0	0	0
	1375	0	0	0	1		486	1	1	1	1
	1467	1	1	1	1		612	1	0	1	1
	2027	0	0	0	1		714	1	1	1	1
	2096	0	0	1	1		738	1	1	1	1
	2125	1	1	1	1		768	1	1	1	1
825	664	1	1	1	1		794	1	1	1	1
	685	1	1	1	1		831	1	1	1	1
	696	1	1	1	1		862	1	1	1	1
	718	0	1	1	1		947	0	0	0	1
	795	0	0	1	0		965	1	1	1	1
	872	1	1	1	1		1250	0	1	1	1
	947	0	0	1	1		1475	0	0	0	1
	1185	1	1	1	1		1886	0	1	0	0
	1202	1	1	1	1		1898	0	1	0	0
	1676	1	1	1	1		2027	0	1	0	0
	1712	1	1	1	1		2046	0	1	0	0
	1975	1	1	1	0						

Genetic similarities among the four Tylophora accessions were estimated according to the ISSR data. At the same time, Jaccard’s coefficient showed that there were two closely related accessions i.e. T_1_ and T_2_ with the highest similarity index (93%). Similarly, the correlation between the time of *in vitro* culture and the extent of genetic instability has been reported earlier in *Picea mariana* and *P. glauca* (Tremblay et al. 1999), *Tectona grandis* (Gangopadhyay et al. 2003) and *Gyposophila paniculate* (Rady 2007). Contrary to the present results, some plant species did not show any genetic changes even after maintained for a period of 26 months, 44 months and 4 years, more than 2 years in *Curcuma longa* (Panda et al. 2007), in *Swertia chirayita* (Joshi and Dhawan 2007), in almond plantlets (Martins et al. 2004) and in *Bambusa balcooa* (Chandrika et al. 2010), respectively. At the same time, Jayanthi and Mandal (Jayanthi and Mandal 2001) reported genetic homogeneity and true-to-type nature of the plants genetic homogeneity and true-to-type nature of the regenerated through somatic embryogenesis of *T. indica*.

**Figure 3:**
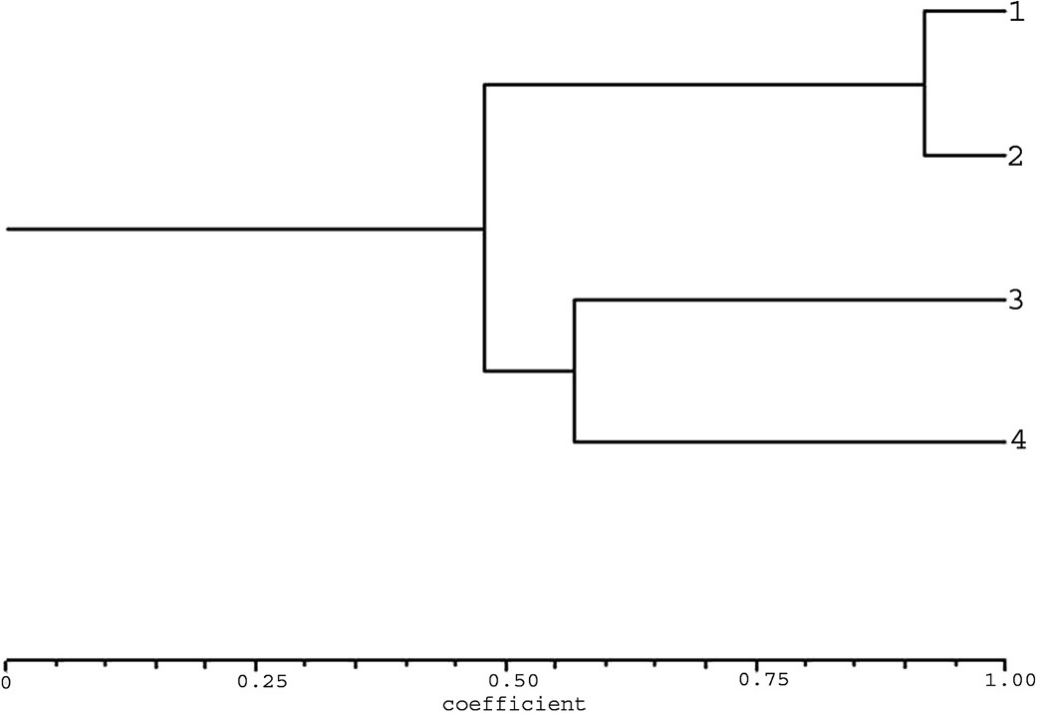
**Consensus tree for four Tylophora accession developed on the basis of their banding patterns with ISSR markers.**

On the other hand, accessions such as T_2_ with T_3_ and T_4_ indicated two most distantly related accessions with low similarity index (57%). The ISSR binary data matrix was followed to calculate Jaccard’s similarity coefficient. Cluster analysis was done via complete linkage method using NTSYS-pc software version 2.02 (Rohlf 2000). Jaccard’s similarity coefficients among the all pair-wise combinations of four accessions based on binary data of six ISSR primers ranging from 0.57 to 0.93 was calculated. A dendrogram generated by cluster analysis using UPGMA method based on Jaccard’s coefficient indicated the genetic similarity of 93% among genotypes from *in vitro* regenerated plantlets through callus organogenesis and the *in vivo* plant from mother plant thus demonstrating the homogeneity of the tissue culture raised plants (Figure 3).

Hence, it becomes essential to check genetic integrity of the micropropagated plants in order to produce clonally uniform progeny while using tissue culture techniques.
